# A Review of Pathogenesis and Risk Factors of Diabetic Retinopathy With Emphasis on Screening Techniques

**DOI:** 10.7759/cureus.31062

**Published:** 2022-11-03

**Authors:** Akarshan Nilay, Archana R Thool

**Affiliations:** 1 Ophthalmology, Jawaharlal Nehru Medical College, Datta Meghe Institute of Medical Sciences, Wardha, IND

**Keywords:** treatment, risk-factors, loss of vision, edema, vegf, duration of diabetes mellitus, diabetic retinopathy

## Abstract

Diabetes mellitus (DM) is an important topic for public health in India and globally. The rise in the number of cases of diabetes in India is alarming as it will eventually increase the number of cases of diabetic retinopathy (DR). DM is related to many microangiopathic abnormalities, among which DR has recently emerged as a common cause of vision impairment in middle and low-income countries. If diagnosed on time, we can prevent vision impairment and complete blindness in patients with the help of proper treatment. Life-long retinal check-ups among people who have diabetes are an essential strategy for the early diagnosis of DR. In this article, we have focused mainly on the significance of DR in loss of vision, the risk factors associated with the disease, the proper pathogenesis of the disease, including the involvement of vascular endothelial growth factor (VEGF), the further consequences of the disease, screening techniques that are already available and those that need to be incorporated, and finally the treatment options available for the patients. The knowledge about this disease and various components related to it, be it either risk factors, pathogenesis, or screening techniques and treatment, would surely help clinicians better understand the condition to formulate strategies for its early diagnosis and cost-effective and sustainable treatment, which would ultimately result in providing better care to the patients.

## Introduction and background

Diabetes mellitus (DM) is a matter of great concern for public health in India. The magnitude of DM has increased in the last five decades in urban and rural India from 3.4% and 2.3%, respectively, in 1973 to 19.2% and 15.1% correspondingly in 2015-2019, which is more than the worldwide urban (10.9%) and rural (7.3%) incidence of DM [1]. The occurrence of DM is very high in middle and low-income countries, which may be due to improving longevity and westernization of lifestyle. Among many microvascular problems of DM, diabetic retinopathy (DR) is the most common and most important reason for preventable blindness globally [2]. Early diagnosis of DR is vital because individuals with DR usually present no signs in the early stage of the condition. It is a retinal vasculitis that occurs as a complication of DM. The ocular changes that usually occur in DR are macular oedema and new vessel formation, the former being the most common complication [3]. There has been a substantial increase in the occurrence of DR, which has helped it become one of the major reasons for blindness and the diminishing of vision in adults above the age of 20 [4].

The earlier stages of DR may not cause severe vision impairment like non-proliferative DR (NPDR). Diabetic macular oedema (DME) and proliferative DR (PDR) are collectively known as vision-threatening DR (VTDR) [5]. If untreated, 26.5% of the population with VDTR is prone to be severely vision impaired in the next 1-2 years [6]. According to the National Diabetic Retinopathy Survey, India, report, during 2015-2019, DR occurred in 17% of patients above the age of 50 years [7]. Timely diagnosis and management of DR can decrease the danger of severe vision loss by up to 90%, which is evaluated by the Early Treatment Diabetic Retinopathy Study (ETDRS) [8]. The fact that has to be taken care of is that diabetes is not detected until the complications of the disease occur. The additional worrisome fact is that most people diagnosed with diabetes never undergo an eye check-up until required [9]. A very complex relationship exists between an individual's socioeconomic status and the disease [10]. Low literacy rates and poverty are the two reasons that usually contribute to poor health-seeking behaviour among people who belong to low socioeconomic status. The unawareness and the delays made by the patients lead to even further complications, eventually increasing the cost of diabetic eye care [11]. Epidemiological studies have suggested a higher prevalence of DM among the "urban poor" and "rural rich," indicating an inverse relationship between socioeconomic status with DM in urban and rural populations [10-12].

## Review

Risk factors

Bad Metabolic Control

A timely and decent glycaemic control might delay and prevent the progress of DR. A higher risk of severity is related to high levels of glycated haemoglobin [13].

Pregnancy

This physiological condition is occasionally associated with rapidly progressing DR [14].

Puberty

There is a very low threat of DR earlier to the onset of puberty, irrespective of the period of diabetes the person suffers from. Once the age of 13 years is attained, the severity and frequency of this disease increase [15]. Hormonal changes might be the responsible factor behind this.

Lipids

It has already been proved that a high level of lipids is related to retinopathy. Elevated levels of hard exudates are associated with high levels of cholesterol. It is the high levels of triglyceride that determine the severity of retinopathy [16].

High Blood Pressure

High blood pressure is one of the most researched systemic factors openly associated with DR. Although it is not clear if the hypertension is because of nephropathy, if so, then both are considered to be diabetic complications [17].

Nephropathy

When different studies were conducted, the coincidence of DR and nephropathy was observed in both type 1 and type 2 diabetes. It is found that DR might be the most common microvascular problem of diabetes, which even precedes nephropathy [18].

Syndrome of Sleep Apnoea

DR and macular oedema can worsen in diabetic patients suffering from sleep apnoea syndrome [19].

Extent of Diabetes

It is undoubtedly the most significant factor. The incidence was 2% in diabetes type 1 patients with no more than two years of progression of diabetes. At the same time, the incidence was found to be 20% in type 2 diabetes patients taking treatment with insulin or without insulin with less than five years of progression of diabetes [20]. It was found to be 80% in patients with 15 years of progression. It is the asymptomatic patients, lack of cost-effective treatment, lack of trained professionals and lack of early diagnosis that lead to an increase in the incidence of type 2 diabetes. DR is very uncommon before puberty.

Irrespective of the above-stated risk factors, readings have suggested an extensive deviation amongst the severity and development of DR that the already identified risk factors cannot entirely clarify. Hence, identifying biomarkers to simplify the danger or assess the healing reaction of DR is vital [21]. Optimum control of all the risk factors mentioned above can benefit people suffering from DM in improving their eye health.

Pathogenesis

**Figure 1:**
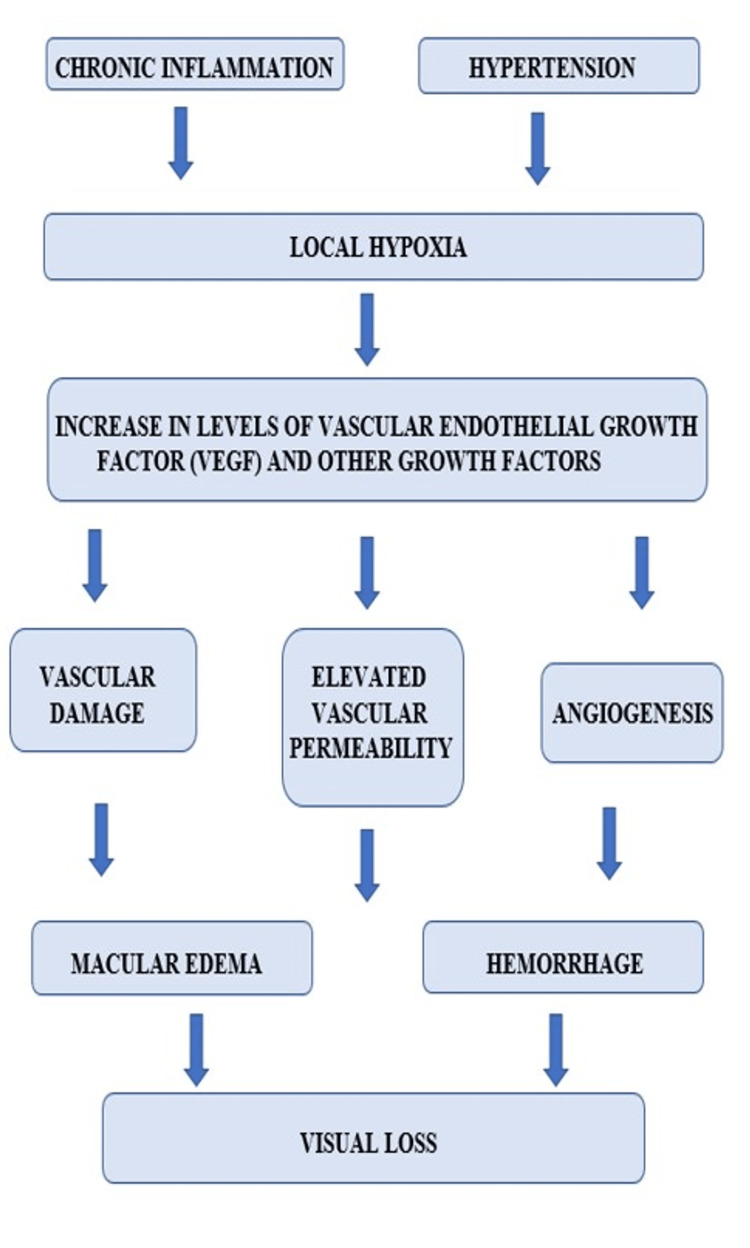
Representation of pathogenesis of diabetic retinopathy

DR is one of the microangiopathies of the retina. It is involved in the variations in the rheological properties and the blood's vascular wall. The incorporation of these factors leads to the obstruction of capillaries and to ischemia of the retina and leakage, which can be identified by angiography. The classic histopathological changes comprise a decrease in endothelial cells and pericytes and solidifying of the basilar membrane. Micro-aneurysms are actually the locations of external swelling of the capillary wall and are pathognomonic [22]. Concerning the rheological properties of the blood, the features that lead to reduced fibrinolysis and raised viscosity of blood are decreased serum albumin concentrations, increased concentrations of fibrinogen and alpha-2 globulin, diminished deformability of erythrocytes, and increased platelet accumulation. The summarization of pathogenesis is shown in Figure 1.

Pathogenesis involves numerous biochemical signal pathways. The amplified action of protein glycosylation and protein kinase C results in the creation of advanced glycation end products (AGE). This is further followed by cell communications comprising vascular endothelial growth factor (VEGF), which leads to new vessel formation in the anterior as well as posterior parts of the eye, the increase in vascular permeability outcomes in a leak, and the breakdown of the inner blood-retina barrier [23]. AGE is formed endogenously in more significant quantities as a reason for hypoglycemia and is also taken exogenously in food items. These appear to intercede almost all difficulties of diabetes together with the inflammatory vessel wall variations and vasoconstriction related to endothelial cells' functioning and macrophages influenced by the development of atheromatous plaques. The goal of the current therapeutic method is the inflammatory vessel wall changes employing the intra-vitreous injection of glucocorticoids [24]. Transforming growth factor β, pigment epithelium-derived growth factor, and Insulin-like growth factors I and II are the other essential factors that have a substantial role in the causation of DR.

Consequences

Retinal damage and hard exudates occur due to the amplified permeability of vessels and drop in levels of lipids and plasma proteins. Micro-thrombosis with retinal micro-infarcts yields cotton wool spots identical to ischemia and hypoxia. Hypoxia yields an effect for discharging angiogenic factors and new vessel construction in the retina and iris. Oedema is produced especially in the macular area by the extravasated fluid [25]. In the circumstances mentioned above, the VEGF is synthesized in various retinal cells, and it may increase by up to 30 times its production in the case of hypoxia [26]. This is possible because of two mechanisms either it stimulates vascular porousness and oedema. As a result, all of the retinal cells change, which might cause vision problems or trigger the creation of new blood vessels. 

Screening

Technically, screening is a procedure of diagnosing an unrecognized disorder or disease with the help of tests in apparently healthy and asymptomatic people. World Health Organization (WHO) conditions for screening programs include: (i) the disorder must represent a significant public health problem, (ii) the disorder must have an identifiable latent/early stage, the screening procedure must be suitable for the public and healthcare professionals, (iii) the disorder must have adequate and well-recognized therapy choices, and (iv) the price of early identification and treatment should be thought about for whole healthcare spending, and the consequence of leaving the disease untreated [27]. It is already known that DR is an asymptomatic disorder that can elevate to permanent loss of vision if not treated timely [28]. The current DR screening guidelines followed in India endorse the initial retinal investigation in patients with type 1 diabetes within five years after identification. Individuals with type 2 diabetes must have their initial retinal investigation at the time of identification of diabetes and, after that, yearly [29]. Frequent retinal examinations are recommended based on the increasing severity of DR. People having diabetes during pregnancy have an amplified risk of developing DR. Approaches to DR screening include indirect as well as stereoscopic fundus photography, direct ophthalmoscopy, and mydriatic or nonmydriatic retinal colour fundus photography [30].

Table 1 describes the numerous strategies that can be used to screen DR. The seven standard field 30° stereoscopic fundus photography, which was established for the grouping and classifying DR using the ETDRS group, is considered the best for diagnosing DR [31]. Most of the existing fundus cameras have a very high resolution that surpasses the 2-3 megapixel resolution, which is essential to present a single micro-aneurysm [32]. By using high-quality images, retinal images can effortlessly be classified by trained optometrists, ophthalmologists, or trained image graders utilizing the International Clinical Classification of Diabetic Retinopathy classification or the ETDRS classification [33]. Retinal colour photography has essential roles in screening, documenting, detecting DR complications, monitoring the progression, counselling the patient, and the response to treatment [34]. Also, mydriatic fundus photography has already been recognized as comparable or even better to the ophthalmoscopic investigation for DR recognition and verified to be the utmost profound screening examination for VTDR [35-36] Since a considerable number of patients who have diabetes need to be screened frequently, a physical grouping of fundus photographs does not seem to be a feasible option. The help of automated DR finding through artificial intelligence can empower immediate identification and be valuable for telemedicine and remote DR screening [37].

**Table 1:** Devices for diabetic retinopathy screening in India

Device	Advantages	Disadvantages
Direct Ophthalmoscope	Relatively inexpensive: easy to carry and easy to use	Needs dilatation for better view; smaller field of view, lower sensitivity, and close contact risk
Indirect Ophthalmoscope	Wider field of view, relatively inexpensive	Mydriasis mandatory, needs training for use/used only by trained ophthalmologists
Slit‑lamp biomicroscopy with contact (three mirror lens) or noncontact lens (78D/90D lens)	Wider field of view, good sensitivity	Mydriasis mandatory, needs training for use/use only by trained ophthalmologists
Nonmydriatic camera Without dilatation	40‑45° field of view; easy to use with minimal training; undilated pupils; can be transported to the community for remote and rural screening in mobile units; linked to the system for electronic data storage	Poor image quality especially for older people with media opacities like cataract Single field image may miss lesions in other field (at least two fields (disc centered and macula centered for each eye to be taken)
With dilatation	Better image quality, imaging of more fields possible. Useful for telemedicine	Good internet connection needed for image transfer for remote grading by trained graders or ophthalmologists
Handheld fundus camera/ smartphone‑based fundus camera	Easy to handle, portable, less expensive; can be charged and then used without electrical power connection; can be connected with WiFi for use in tele‑ophthalmology/rural screening; can have AI‑integrated automated system	Image quality may be inferior without mydriasis especially for older people with cataract. Mydriasis improves gradability. Good internet connection needed for transfer of images for remote grading
Conventional desktop camera	Larger field of view (50°); excellent resolution: seven field stereo photography is possible: easy to use with training by optometrists; good image quality; used especially in the eye hospitals	Mydriasis required, expensive
Ultrawide field fundus camera (Scanning Laser Ophthalmoscope)	Very wide field of view up to 200° without dilatation; can detect peripheral DR lesions also	Very expensive, cannot be used for mass screening in India unless the cost gets reduced

Efforts to escalate the screening for DR must be supplemented by efforts to raise awareness and knowledge related to diabetes and its problems, especially DR, to confirm regular follow-up of the patients [38]. Various readings have presented that the enactment of regular DR screening programs at a national level in a few developed nations has decreased the cases of visual loss caused due to DR [39]. Table 2 shows the numerous models of DR screening being used and also those that can be useful for India.

**Table 2:** Various screening models for diabetic retinopathy

Screening model Techniques		
A	Hospital-based screening model screening by physicians/diabetologists (complications screening by diabetologist)	
	Primary physician clinic	Examination by physician with a direct ophthalmoscope
	Secondary care hospital/multispecialty polyclinics	1. Referral to a trained ophthalmologist for fundus examination, or 2. Use of nonmydriatic fundus camera for retinal imaging by trained eye technician/optometrists
	Tertiary care hospitals/diabetology/ endocrinology clinics	1. Referral to a retina specialist for fundus examination and management, 2. Fundus photography: grading of fundus photographs by trained graders/ophthalmologists and/or artificial intelligence (AI)
	Ophthalmologist‑based screening tertiary care eye facilities	1. Fundus examination by trained ophthalmologist with indirect ophthalmoscopy or slit‑lamp biomicroscopy, 2. Fundus photography: grading of fundus photographs by trained optometrists/ophthalmologists
B	Community‑based screening model diabetes screening programs, rural outreach programs, at pharmacies/optical shops, risk‑based screening	Use of nonmydriatic fundus camera for retinal imaging by trained eye technician and images graded by trained graders or trained ophthalmologists
C	Teleophthalmology screening model	Use of nonmydriatic fundus camera for retinal imaging by trained eye technician and images graded by trained graders or trained ophthalmologists or with use of artificial intelligence

Hospital-based DR screening model

Physician Clinics

Regular physicians competent in utilizing a direct ophthalmoscope can observe the fundus of patients who have diabetes. Physicians are usually the initial interaction for people who have diabetes. Various diabetologists and physicians throughout the country have undertaken training for fundus investigation by the Public Health Foundation of India (PHFI), a certificate program of DR for increasing the number of optometrists as well as physicians. Sometimes physicians fear dilating the people for fundus inspection [40]. Unfortunately, without dilatation, the diabetologist or physician can only observe the optic disc or macula. In older people, the investigation is more challenging due to age-related cataracts. While remaining asymptomatic, patients with DM are usually inclined to decline a dilated fundus inspection due to the absence of information that diabetes can also affect their eyesight. Direct ophthalmoscopy fundus examination involves approaching to close vicinity of the patient; therefore, it might be thought-provoking, particularly with the continuing coronavirus disease 2019 (COVID-19) pandemic. DR screening and inter-referral can be performed in polyclinics or hospital set-ups so the diabetologists can send patients with diabetes to the ophthalmologists who are employed in the same hospital, and the ophthalmologists can send patients with DR to the diabetologists for glycaemic control.

Screening at Non-Communicable Disease (NCD) Clinics

A wide-ranging platform has been started in public health NCD clinics in almost 10 regions throughout India to identify eye problems among individuals with diabetes [41]. A nationwide task force was organized to supervise strategy formation and deliver a planned track to the program [41]. District and state organization committees were formed to supervise the growth of the planned program. Using non-mydriatic cameras, 66,456 people having diabetes were examined in those set-up clinics. A total of 10,766 (16.3%) individuals were observed to have DR, among which 4975 (7.54%) had VTDR. Approximately 80.9% of people with VTDR were successfully cured [41]. The research conveyed that there was a sevenfold growth in DR screening through the use of this platform. The scheme presented that a combined tactic can empower effective DR recognition as well as management at sub-district and district stages.

Hospitals and Dispensaries

Secondary care hospitals that have fundus cameras employ skilled eye experts for fundus imaging to screen patients with diabetes coming to their hospitals. Patients who have diabetes who went to several dispensaries under the Mumbai Municipal Corporation were examined for DR with the help of a mobile phone-based retinal camera by a trained healthcare worker. The pictures were categorized by a trained ophthalmologist as well as an AI system. The accuracy of the AI system in analyzing DR was almost 100%, and the specificity was 88.5%. The research displayed that the help of an automated offline algorithm integrated with a low-cost fundus camera could be an upgradable mode of DR screening in India [42].

Tertiary Care Diabetes Centres

Screening for DR is achievable predominantly in tertiary care diabetes centres as the patients with inadequate glycaemic control show up to those centres. Also, yearly follow-up appointments are more feasible in such establishments. Usually, patients with diabetes do not come to the ophthalmologist for DR screening because of a lack of awareness about the fact that eyes too can be affected by diabetes [43]. The importance of diabetes educators and diabetologists in improving awareness about retinal inspection amongst patients is vital.

Multi-Speciality Eye Care Facilities and Eye Hospitals

Competent ophthalmologists usually investigate DR using slit lamp bio-microscopy with the help of a 78 D/90 D lens and indirect ophthalmoscopy for a complete retinal check-up following dilatation of the pupil (mydriasis). Tertiary eye care centres provide fundus investigation knowledge and disease management with well-equipped facilities. All patients with diabetes coming to eye hospitals for a comprehensive eye test or refraction can be sent to the speciality retina clinic for high-value retinal imaging following mydriasis for precise DR identification and reasonable control of VTDR. On the other hand, the low number of eye hospitals and competent specialists is the obstacle to the widespread execution of such screening. To lessen the load on ophthalmologists, professional fellowship courses are being offered to increase the number of optometrists. To conclude, we can say that hospital-based screening programs are more specific and empower screening and DR findings in identified diabetes patients [44].

Treatment

Despite optimal medical management of serum cholesterol level, blood glucose level, and blood pressure, several intraocular procedures have developed as regular treatments for DR [45]. In the case of patients with DR, anti-VEGF treatment has improved the treatment comprising drugs such as bevacizumab, aflibercept, and ranibizumab. Various large-scale randomized trials have verified the effectiveness of all the above-mentioned anti-VEGF drugs in lessening diabetic macular oedema and improving vision [46]. Many patients might need a regular injection of intra-vitreous anti-VEGF throughout the initial year of therapy, and lesser injections are required in subsequent years for the upkeep of remission. Panretinal laser photocoagulation is more effective in reducing the danger of vision loss in proliferative diabetic retinopathy patients. Recent studies have shown that intravitreal injection of anti-VEGF might be a harmless therapy for PDR. However, adherence to treatment burdens repeated follow-up, and patients’ inclinations should be considered while using the clinical trial outcomes in clinical practice in the real-world scenario. The molecular origin of DR is under observation. These readings may assist in the finding of novel drugs and deliver more customized therapies [47].

## Conclusions

DR is a complication of DM. Even after various studies concerning the management modalities of DR and macular oedema, the limitation of proper medical resources resulted in a low fundus examination rate, which has delayed the identification and timely management of DR. On average, there is a five-year gap between the onset of symptoms and diagnosis of the disease, which should be used appropriately to prevent further progression. Hence, applying an automated screening technique and identifying specific biomarkers is vital for early diagnosis and proper treatment.
